# Post-Initiation Care Patterns and Weight Loss With Semaglutide and Tirzepatide for Obesity in Clinical Practice

**DOI:** 10.64898/2026.10.04.26364715

**Published:** 2026-10-06

**Authors:** Huanhuan Yang, Chungsoo Kim, Joseph S. Ross, Chenxi Huang, Kyungseon Choi, Bo Kang, Adith S. Arun, Huilin Tang, Yong Chen, Hua Xu, Mona Sharifi, Zhihui Li, Harlan M. Krumholz, Yuan Lu

## Abstract

**Background:**

Randomized trials show substantial weight loss with semaglutide and tirzepatide under structured care, but little is known about whether patients in routine practice continue therapy, reach a maintenance dose, and have their weight measured.

**Objectives:**

To describe the first year after initiation of semaglutide or tirzepatide for obesity, including care patterns, maintenance-dose attainment, weight documentation, and total body weight loss (TBWL).

**Methods:**

This retrospective cohort study used Epic Cosmos, an electronic health record network of >350 US health systems, to identify adults with obesity and without diabetes who initiated injectable semaglutide or tirzepatide in 2024. Outcomes over 12 months included prescribing persistence, discontinuation, switching, add-on therapy, and reinitiation; attainment of Food and Drug Administration (FDA)-labeled maintenance doses; availability of follow-up weight; and TBWL across progressively restricted populations.

**Results:**

Among 743,153 adults (60.1% semaglutide; mean age, 48.5 years; 72.5% female), 12-month prescribing persistence was 29.9% for semaglutide and 35.9% for tirzepatide. FDA-labeled maintenance-dose attainment was 28.3% and 55.0%, respectively, and only 8.8% and 16.4% remained prescribing persistent at a maintenance dose with a recorded weight at 12 months. About half of initiators lacked a 12-month weight. Among all initiators, ≥5% TBWL was documented in 25.6% (semaglutide) and 30.4% (tirzepatide), rising to 51.9% and 61.9% among those with recorded weight, 66.1% and 79.5% among those also prescribing persistent, and 82.9% and 87.3% among those further at a maintenance dose.

**Conclusions:**

In routine practice, most patients initiating semaglutide or tirzepatide did not remain on therapy, reach a maintenance dose, and have weight documented through the first year. Documented weight loss was greatest among those completing each step of care, suggesting continuation, dose escalation, and monitoring as targets for improvement.

**CENTRAL ILLUSTRATION:**

Care Cascade and Weight-Loss Outcomes Among Semaglutide and Tirzepatide Initiators, Standardized to 100 Initiators.

Numbers are per 100 initiators and were rounded so that categories at each step sum to the preceding step. At the 12-month dose step, prescribed dose was missing for 1 per 100 semaglutide initiators and <1 per 100 tirzepatide initiators.

## Full Text Availability

The license terms selected by the author(s) for this preprint version do not permit archiving in PMC. The full text is available from the preprint server.

